# Mapping research trends in irritable bowel syndrome and the gut microbiome: a cross-database bibliometric analysis

**DOI:** 10.3389/fmicb.2026.1900159

**Published:** 2026-07-22

**Authors:** Weijiu Mo, Fuqing Cai, Liwei Li, Jingrong Liang, Jinxiu Zhang, Li Yao, Sujuan Mo, Yanyan Liao, Sufan Tang, Zheng Liu, Zhiliang Chen, Mengbin Qin, Shiquan Liu, Jun Zou, Jiean Huang

**Affiliations:** Department of Gastroenterology, The Second Affiliated Hospital of Guangxi Medical University, Nanning, Guangxi, China

**Keywords:** bibliometric analysis, dysbiosis, gut microbiome, gut–brain axis, irritable bowel syndrome, short-chain fatty acids

## Abstract

**Background/objectives:**

Research on irritable bowel syndrome (IBS) and the gut microbiome has expanded rapidly. However, the structural evolution of this literature has not been systematically characterized across major indexing platforms.

**Methods:**

We performed a parallel bibliometric analysis of the Web of Science Core Collection (WoSCC, *n* = 1,502), Scopus (*n* = 1,163), and PubMed (*n* = 975). The analysis included English-language articles and reviews published from January 2000 to December 2025. WoSCC served as the primary dataset, and Scopus and PubMed were analyzed in parallel for cross-database comparison. Bibliometric mapping and visualization were performed using VOSviewer, CiteSpace, and bibliometrix.

**Results:**

Annual output increased from fewer than 10 articles per year before 2010 to 162 in 2025. This acceleration became marked after 2014 and was reproduced across all three databases. The United States and China led publication volume, whereas the UK output was concentrated in a small number of flagship centers. Our analysis suggests three developmental phases: compositional profiling and culture-dependent benchmarks (2000–2012), community-level characterization and interventional consolidation (2012–2017), and neuroendocrine and short-chain fatty acid mechanisms (2017–2025). Visceral hyperalgesia and hypothalamic–pituitary–adrenal axis dysregulation showed the strongest currently active keyword bursts. Diet-related and precision-oriented approaches also gained visibility.

**Conclusion:**

IBS–gut microbiome research has shifted from descriptive profiling toward mechanistic, diet-related, and precision-medicine themes. These findings provide a structured overview of the field and help clarify priorities for its next phase.

## Introduction

Irritable bowel syndrome (IBS) is among the most prevalent disorders of gut–brain interaction worldwide. Under the Rome IV criteria, the pooled adult prevalence is approximately 4 to 5%, with higher estimates under older diagnostic definitions (Sperber et al., 2021; Black and Ford, 2020; Oka et al., 2020). IBS also imposes a substantial clinical and economic burden. This burden arises from chronic symptoms, frequent psychiatric comorbidity, repeated diagnostic evaluations, and sustained use of ambulatory care (Black and Ford, 2020; Canavan et al., 2014). Health-related quality of life in IBS is comparable to that reported in inflammatory bowel disease or diabetes. In most health-system analyses, indirect costs from work absenteeism and productivity loss exceed direct medical costs (Buono et al., 2017; Ford et al., 2020). These features give IBS major public health relevance, yet until comparatively recently its pathophysiological basis remained poorly defined (Black et al., 2025).

For much of its clinical history, IBS was defined by absence. Structural pathology was not detected, biochemical markers were unavailable, and tissue damage could not be demonstrated on conventional endoscopy or histology (Holtmann et al., 2016). Diagnostic frameworks therefore evolved through successive iterations of symptom-based criteria, including Manning and Rome I–IV. These criteria improved clinical recognition but offered limited mechanistic insight (Lacy et al., 2016). Traditional models emphasized visceral hypersensitivity, altered motility, and psychological stress. Although these models captured important dimensions of IBS, they did not explain its heterogeneity in symptoms, treatment response, and long-term course (Ford et al., 2020; Enck et al., 2016). The absence of a mechanistic framework also constrained therapeutic development, leaving the field dependent on symptomatic agents with modest and variable efficacy (Camilleri, 2021).

The emergence of culture-independent microbial profiling has transformed this landscape. Sequencing studies have documented reproducible compositional differences between IBS patients and healthy controls (Rajilić–Stojanović et al., 2011; Malinen et al., 2005), post-infectious cohorts have shown that external microbial disruption can initiate durable symptomatic states (Spiller and Garsed, 2009; Sundin et al., 2015), and mechanistic studies have established bidirectional communication among the intestinal microbial community, the enteric nervous system, and the central stress-response axis (Collins et al., 2012; Cryan et al., 2019). The 2016 Rome IV classification, which reconceptualized IBS as a disorder of gut–brain interaction, formalized this shift (Lacy et al., 2016). Trials of probiotics, antibiotics, prebiotics, and dietary manipulation have similarly converged on the microbiota as a modifiable target (Ford et al., 2018; Pimentel et al., 2011; Halmos et al., 2014; Lacy et al., 2021). More broadly, lifestyle-related exposures are increasingly recognized as modulators of the gut microbial ecosystem, with lifestyle-associated dysbiosis linked to inflammatory, oxidative-stress, metabolic, and immune-related pathways (Zhou et al., 2026). These observations extend the rationale for examining microbiota-directed and lifestyle-sensitive mechanisms in IBS. Nevertheless, key questions remain unresolved. The causal direction of dysbiosis in IBS is still contested (Liu et al., 2023). Microbial signatures do not map cleanly onto symptom-based subtypes (Jeffery et al., 2012). Therapeutic responses also show substantial inter-individual heterogeneity (Ford et al., 2018; Ford and Moayyedi, 2010; Su et al., 2023), and translation of mechanistic discoveries into stratified clinical trial designs has lagged behind the accumulation of observational evidence (Ford et al., 2020; Lacy et al., 2021; Ghaffari et al., 2022).

Given the scale and heterogeneity of this literature, narrative reviews and conventional systematic reviews alone are no longer sufficient to map the field’s structure and dynamics. Bibliometric analysis offers a complementary quantitative lens for characterizing the architecture and evolution of a research domain at scale. Traditional systematic reviews are well suited to synthesizing evidence around predefined clinical or mechanistic questions. In contrast, bibliometric methods examine how a field is organized, how its intellectual structure changes over time, and which authors, institutions, publications, and topics shape its development. These methods can quantify collaboration networks, citation structures, keyword evolution, thematic migration, and emerging research fronts (Donthu et al., 2021). They are therefore particularly useful for a rapidly expanding field such as IBS–gut microbiome research, where studies span multiple disciplines, analytical methods, therapeutic concepts, and indexing platforms. In doing so, they can reveal structural patterns that narrative synthesis cannot reliably capture (Aria and Cuccurullo, 2017; Van Eck and Waltman, 2010).

A previous bibliometric analysis mapped the microbiome–gut–brain axis through 2018 (Zyoud et al., 2019). However, IBS–gut microbiome research has not been characterized across a longer time span and multiple databases. Reliance on a single indexing platform may introduce selection bias because the major databases differ systematically in journal coverage, regional representation, and indexing conventions (Mongeon and Paul-Hus, 2016). A multi-database design using identical analytical pipelines applied to WoSCC, Scopus, and PubMed in parallel therefore offers a methodologically stronger basis for characterizing a rapidly evolving field such as IBS–microbiome research, and in particular allows one to distinguish substantive structural patterns from platform-specific indexing effects.

We therefore conducted a cross-database bibliometric analysis of the IBS–gut microbiome literature from 2000 to 2025 using WoSCC as the primary analytical dataset, with Scopus and PubMed queried in parallel as independent validation platforms. Our specific objectives were to: (1) characterize the temporal, geographic, and institutional architecture of the field, including article-type and impact-factor stratified patterns; (2) map the thematic evolution of the knowledge base through keyword co-occurrence networks and reference- and keyword-level citation burst detection; (3) identify structurally pivotal publications and emerging research fronts across the field’s three developmental phases; and (4) critically appraise how the current literature has approached the field’s unresolved questions and derive concrete, actionable directions.

## Materials and methods

### Data collection and search strategy

For this bibliometric study, we searched three major citation databases: the Web of Science Core Collection (WoSCC), Scopus, and PubMed. The search captured publications addressing the association between irritable bowel syndrome and the gut microbial ecosystem from 1 January 2000 to 31 December 2025. This tri-database design reflects the complementary strengths of the three platforms: WoSCC offers high-quality journal coverage with robust citation metadata supporting local-citation-based burst analysis. Scopus provides broader journal coverage, including clinical and nutrition titles that are not consistently indexed in WoSCC. PubMed offers comprehensive coverage of biomedical literature with controlled MeSH vocabulary (Mongeon and Paul-Hus, 2016; Falagas et al., 2008; Kokol and Blažun Vošner, 2018). WoSCC was designated as the primary dataset for all principal bibliometric analyses. Scopus and PubMed were queried in parallel as independent validation platforms.

The keyword framework combined disease-specific descriptors with a deliberately broad set of microbial ecology terms. Disease-side terms (“irritable bowel syndrome,” “IBS,” and the proximity-based expression of the variant term irritable bowel) were constructed to ensure capture of both current and legacy clinical terminology while avoiding false positives from unrelated bowel disorders. Microbiome-side terms were designed to span the field’s full methodological vocabulary, from classical community descriptors (microbiota, microbiome, gut flora, intestinal flora, dysbiosis) to molecular and methodological terms (16S rRNA, shotgun sequencing, metagenome, metataxonome, metatranscriptome, microbial community, microbial ecology) and domain-adjacent compartments (mycobiome, virome, phageome). This framework was developed iteratively through pilot searches, and it balances sensitivity (to avoid omission of relevant studies) against specificity (to exclude studies in which the microbiome is a peripheral rather than a substantive topic).

The WoSCC search was implemented in the Topic field using the string TS = ((“irritable bowel syndrome” OR IBS OR (irritable NEAR/2 bowel)) AND (microbiom* OR microbiot* OR “gut flora” OR “intestinal flora” OR dysbios?s OR metagenom* OR metataxonom* OR “microbial communit*” OR “microbial ecology” OR “16S rRNA” OR “shotgun sequenc*” OR mycobiom* OR virome* OR phageome)). The Scopus search was run in the TITLE-ABS-KEY field using an equivalent query with the W/2 proximity operator in place of NEAR/2. The PubMed strategy combined MeSH descriptors with title and abstract keywords: (“Irritable Bowel Syndrome”[Mesh] OR “irritable bowel syndrome”[tiab] OR IBS [tiab] OR (irritable [tiab] AND bowel [tiab])) AND (microbiom*[tiab] OR microbiot*[tiab] OR “gut flora”[tiab] OR “intestinal flora”[tiab] OR dysbiosis [tiab] OR dysbioses [tiab] OR metagenom*[tiab] OR metataxonom*[tiab] OR “microbial communit*”[tiab] OR “microbial ecology”[tiab] OR “16S rRNA”[tiab] OR “shotgun sequenc*”[tiab] OR mycobiom*[tiab] OR virome*[tiab] OR phageome [tiab]). Proximity operators and MeSH terms were used consistently across databases to minimize platform-specific retrieval artifacts. All database searches were conducted on 1 January 2026, and no additional updates were applied thereafter.

**Figure 1:**
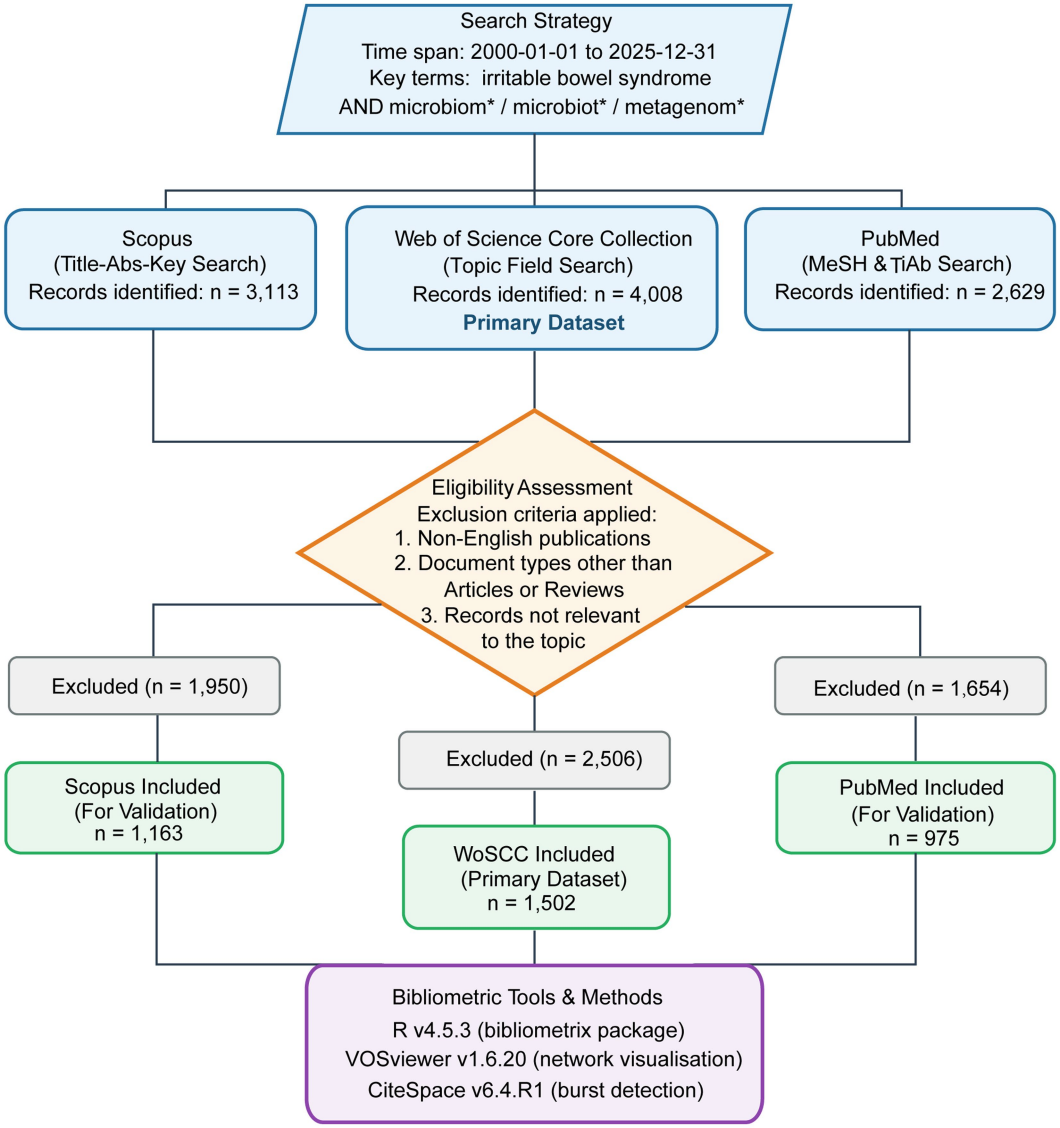
Workflow diagram of literature retrieval, within-database duplicate removal, and screening across three citation databases. Web of Science Core Collection (WoSCC), Scopus, and PubMed were searched in parallel for publications from January 2000 to December 2025 addressing irritable bowel syndrome (IBS) and the gut microbiome. After restriction to English-language original articles and reviews, duplicate removal, and sequential eligibility screening—excluding publications without substantive microbiome-related endpoints, conference abstracts, editorials, case reports, and letters, 1,502 WoSCC records, 1,163 Scopus records, and 975 PubMed records were retained. WoSCC constituted the primary analytical dataset, while Scopus and PubMed served as independent cross-database validation corpora.

Only English-language articles and reviews were eligible for inclusion. Publications focused exclusively on pharmacological mechanisms, drug efficacy, or clinical outcomes without any microbiome-related endpoint were excluded, as were conference abstracts, editorials, case reports, and letters. Duplicate records were removed independently within each database before eligibility screening, using available bibliographic identifiers, including DOI or PMID when available, together with title, publication year, and first-author information. WoSCC, Scopus, and PubMed were then curated and analyzed as parallel datasets. WoSCC was prespecified as the primary analytical dataset because of its standardized citation metadata, while Scopus and PubMed served as independent validation corpora. The three database searches initially returned 4,008 records in WoSCC, 3,113 in Scopus, and 2,629 in PubMed. After applying the eligibility criteria, removing duplicates, and screening titles and abstracts, 1,502 records were retained in WoSCC, 1,163 in Scopus, and 975 in PubMed for analysis (Figure 1).

Journal impact factors and quartile classifications were obtained from the 2024 edition of Clarivate’s Journal Citation Reports (JCR). This research did not include experiments involving humans or animals; therefore, ethical approval and informed consent were not applicable. Given the bibliometric nature of the study, prospective registration was not applicable. This work adhered to the TITAN Guidelines 2025 regarding the disclosure and use of artificial intelligence in scholarly publishing (Agha et al., 2025).

### Bibliometric analysis

Bibliometric profiling followed a multi-tool workflow that combined quantitative description, network visualization, and thematic evolution mapping. Collaboration and conceptual structures were mapped with VOSviewer v1.6.20 using fractional counting, with minimum thresholds of five co-occurrences per keyword. CiteSpace (v6.4.R1) was applied to detect citation bursts of references and keywords across annual slices covering the period from 2000 to 2025. These analyses identified influential publications and evolving research hotspots. Data processing and visualization were carried out in R (v4.5.3) using bibliometrix and several supporting packages, including readxl, dplyr, scales, tidyr, stringr, countrycode, and ggplot2. Multiple-country publication (MCP) ratios were used to describe international collaboration at the country level. R scripts used for bibliometrix-based processing, statistical testing, and visualization, together with the CiteSpace and VOSviewer parameter configurations, are available from the corresponding author upon reasonable request. Author impact indicators reported herein, including the h-index, were derived exclusively from the retrieved dataset and therefore represent local rather than global metrics of scholarly influence.

### Statistical analysis

As bibliometric variables (e.g., author numbers, reference counts, and citation frequencies) generally showed right-skewed distributions, nonparametric statistical methods were adopted throughout the study. Two-group differences were examined using two-sided Wilcoxon rank-sum tests, while comparisons among three or more groups were assessed by Kruskal–Wallis tests followed by pairwise Wilcoxon *post hoc* analyses. Multiple-testing correction was performed using the Benjamini–Hochberg procedure to control the false discovery rate. Continuous variables are reported as median (interquartile range), with statistical significance set at a two-sided *α* value of 0.05. Exact binomial tests with BH adjustment were additionally conducted for one-sample proportion analyses across countries when assessing deviations from predefined null proportions. All computations were conducted in R version 4.5.3.

## Results

### Retrieval, screening, and corpus composition

The database searches returned 4,008 records in WoSCC, 3,113 in Scopus, and 2,629 in PubMed (Figure 1). After restricting to English-language original articles and reviews and applying the eligibility criteria described in Section 2.1, 1,502 WoSCC records were retained for primary bibliometric analyses, while 1,163 Scopus records and 975 PubMed records were retained for cross-database comparison. The final WoSCC corpus comprised 1,040 research articles and 462 reviews, while Scopus contained 798 research articles and 365 reviews.

### Annual publication output

**Figure 2:**
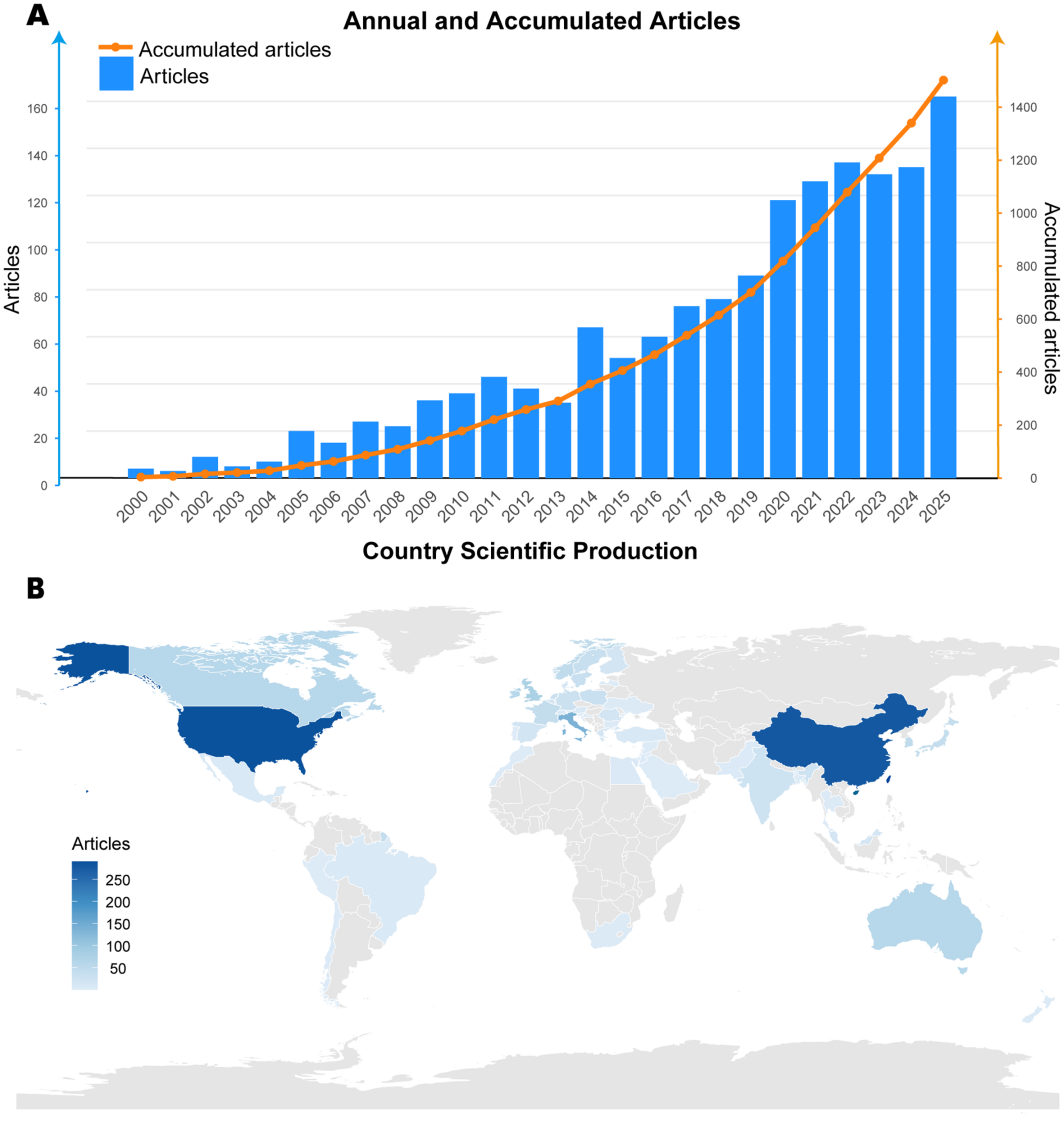
Temporal trends and global geographic distribution of publications related to IBS and the gut microbiome. **(A)** Annual and cumulative publication outputs indexed in the Web of Science Core Collection from 2000 to 2025, demonstrating sustained growth in research activity, particularly after 2014. **(B)** Global scientific output by country or region. Color intensity represents publication volume, with darker colors indicating higher research productivity. The United States and China were among the leading contributors.

Annual output in the WoSCC corpus rose from fewer than 10 articles per year during 2000–2009 to 162 articles in 2025 (Figure 2). Three successive developmental phases were evident: an early phase of modest and relatively flat output (approximately 2000–2012), a transitional phase of steadier growth (approximately 2012–2017) and a rapid expansion phase from 2017 onward that accounted for 53.3% of the cumulative WoSCC corpus. The Scopus (*n* = 1,163) and PubMed (n = 975) series displayed the same overall temporal structure, with parallel acceleration from approximately 2014 and a modest plateau–recovery pattern after 2022 (Supplementary Figures 1 and 2).

### Article types and study characteristics

**Figure 3:**
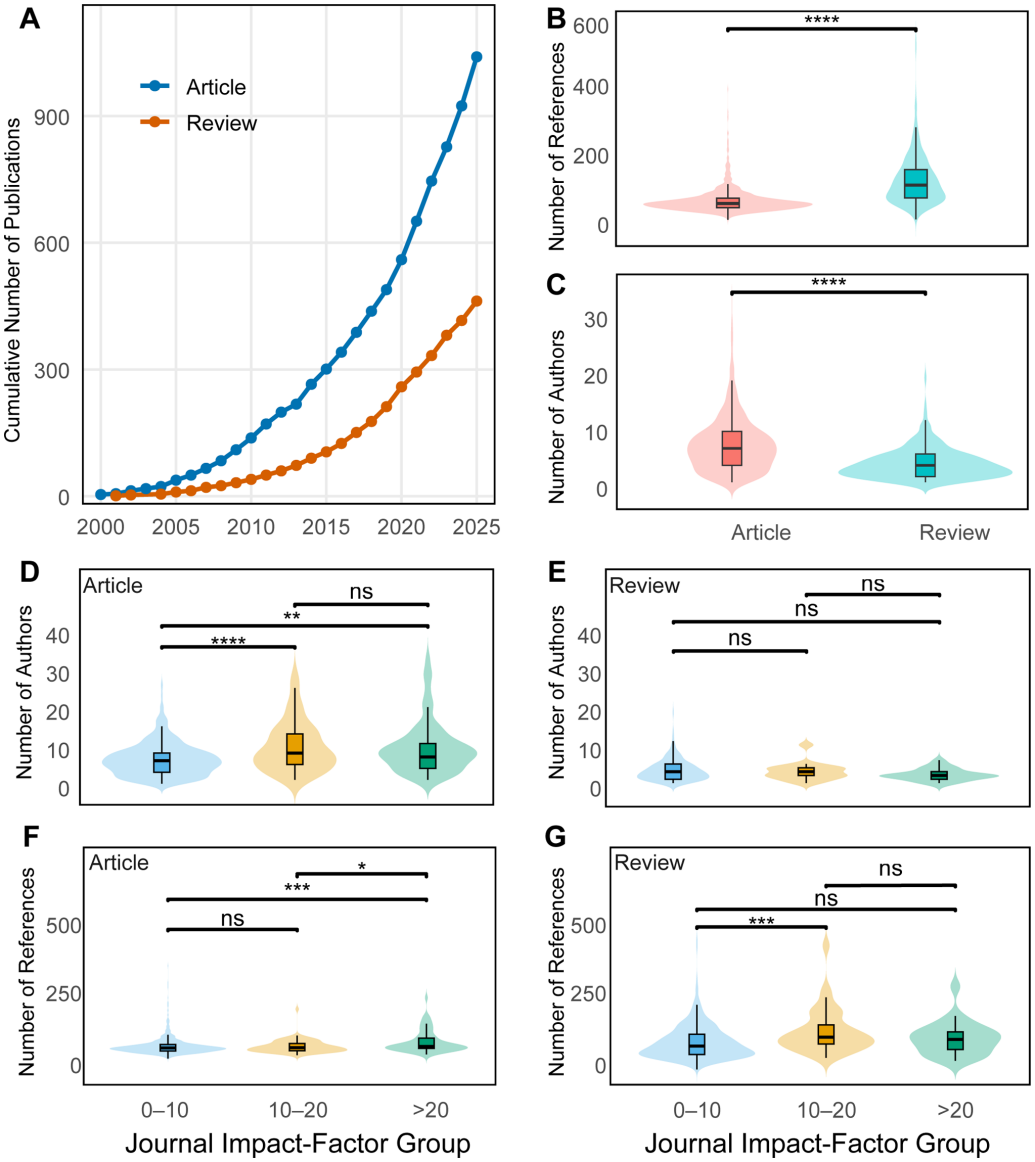
**(A)** Cumulative publication trends comparing research articles and review articles within the dataset. **(B,C)** Comparison of reference counts and author team sizes by document type. Boxplots show that research articles tend to have larger author teams, while review articles include more references. **(D,E)** Author team size distributions across journal impact factor categories for research and review articles, indicating differences in collaboration scale across journal tiers. **(F,G)** Reference count distributions across impact factor categories, reflecting variation in citation density with journal prestige. Significance levels from pairwise Wilcoxon tests with Benjamini–Hochberg correction are indicated as: * *p < 0.05*; ** *p < 0.01*; *** *p < 0.001*; **** *p < 0.0001*; ns, not significant.

The WoSCC corpus comprised 1,040 research articles and 462 reviews (Figure 3A). Reviews cited substantially more references (*p < 0.0001*; Figure 3B), whereas research articles involved significantly larger author teams than reviews (*p < 0.0001*; Figure 3C). When stratified by journal impact factor (IF), team size in research articles expanded sharply between the IF 0–10 and IF 10–20 tiers (*p < 0.0001*) and then plateaued, with no significant difference between the IF 10–20 and IF > 20 groups (Figure 3D). Review-article team size showed no significant variation across any IF tier (Figure 3E). Reference counts in research articles showed no significant difference between the IF 0–10 and IF 10–20 tiers, but increased significantly in the IF > 20 tier (Figure 3F). Review articles demonstrated a different pattern, with reference counts rising significantly from the IF 0–10 to the IF 10–20 tier and then plateauing, with no further increase in the highest IF group (Figure 3G). The Scopus analyses reproduced the article-type difference in authorship scale, with research articles consistently attracting larger teams than reviews, but did not replicate the IF-stratified expansion in research-article team size observed in WoSCC, suggesting that the coupling between authorship scale and journal impact is partially database-dependent (Supplementary Figure 3).

### Country-level contributions and international collaboration

**Figure 4:**
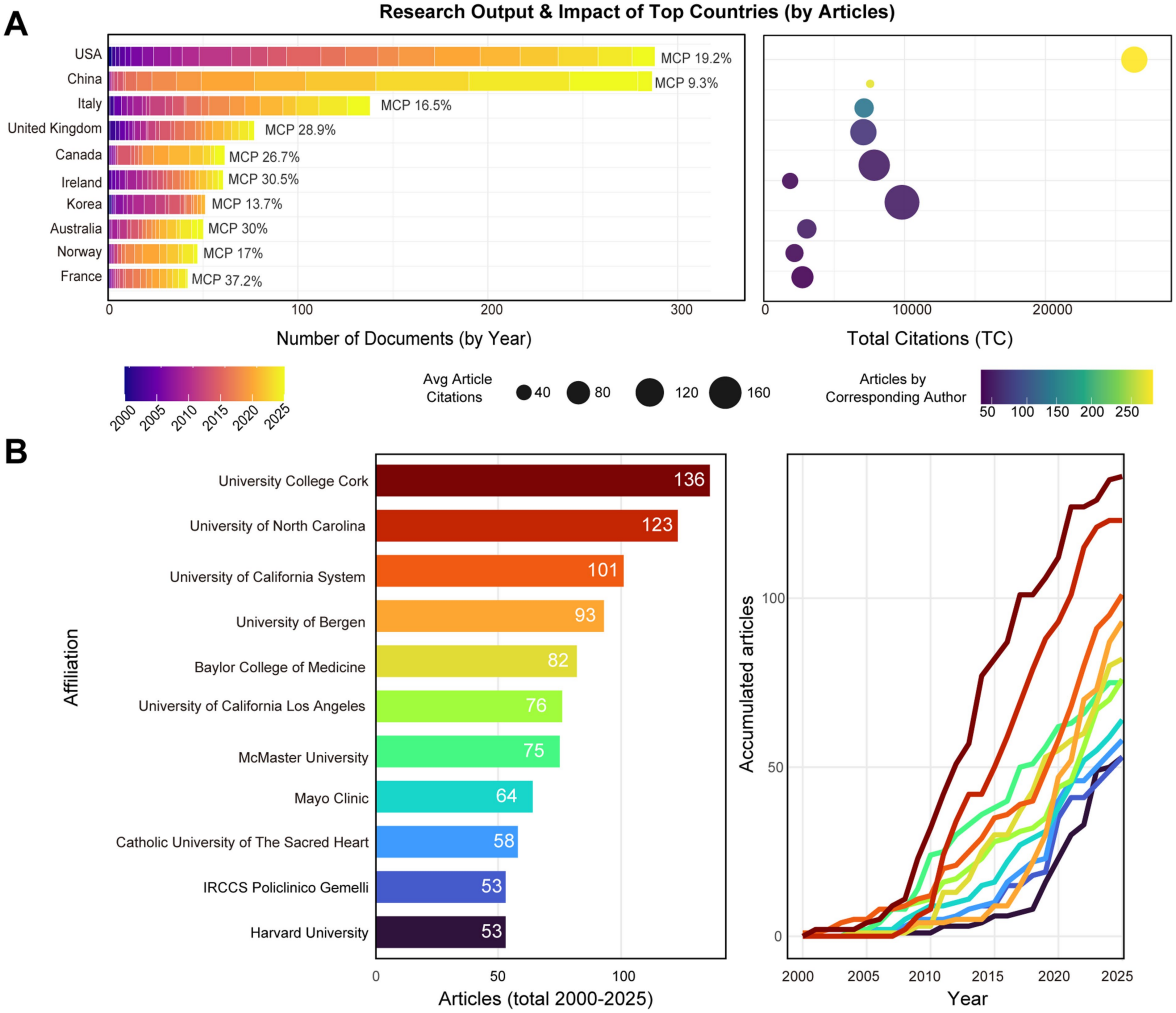
Scientific output and impact of top contributing countries and institutions in IBS–gut microbiome research. **(A)** Top 10 countries ranked by publication output. Total publications are shown alongside indicators of international collaboration (MCP) and citation impact. **(B)** Publication trends and cumulative output of the top 11 institutions, showing changes in productivity over time and the emergence of leading research centers in the field.

**Figure 5:**
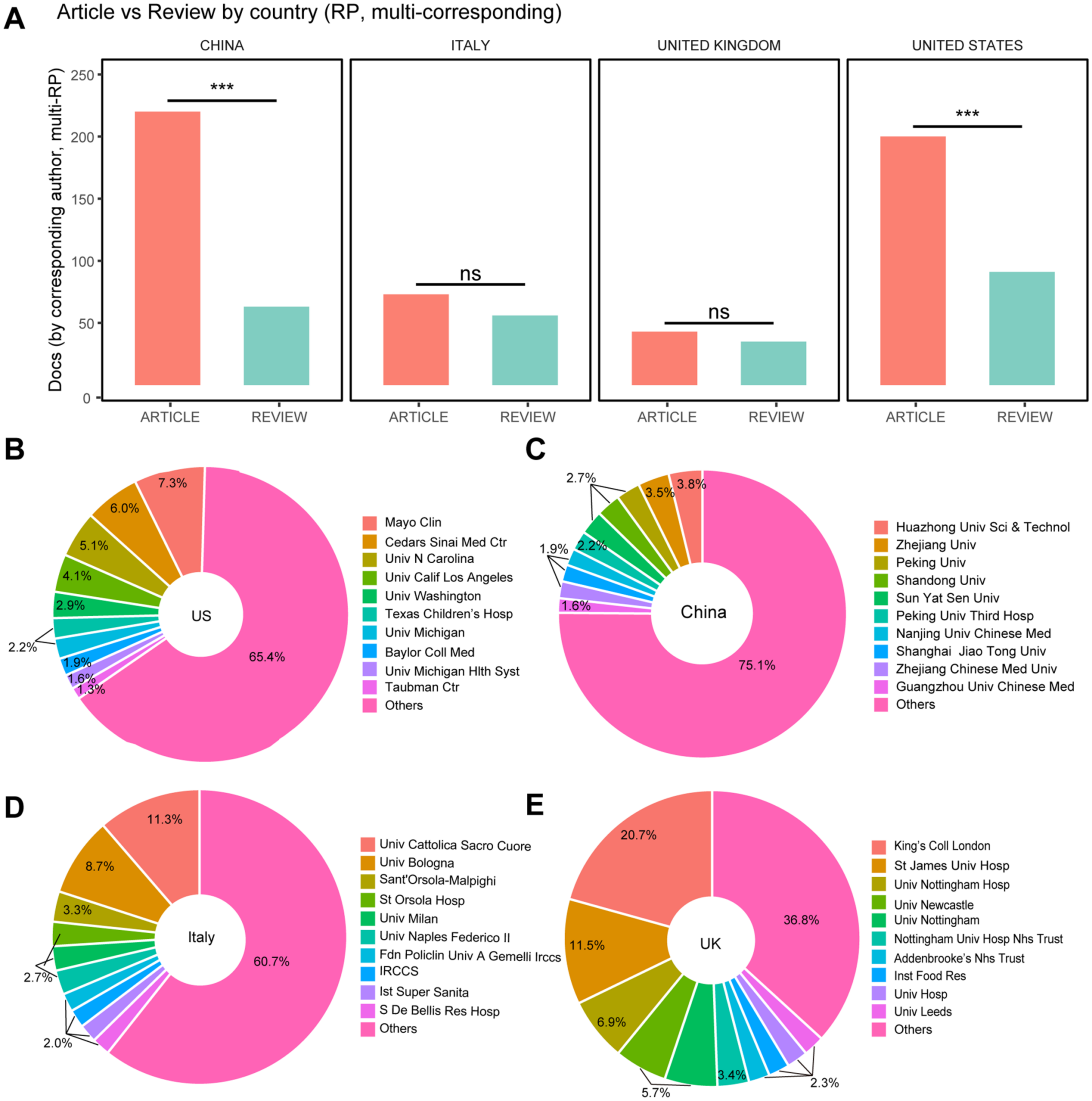
Article-type composition and institutional concentration of research output among the four leading contributing countries. **(A)** Comparison of the number of original articles and reviews published with multiple corresponding authors in China, Italy, the United Kingdom, and the United States. Statistical significance was assessed using the Wilcoxon rank-sum test; ***p < 0.001; ns, not significant. **(B–E)** Donut charts illustrating the institutional distribution of multi-RP publications in the United States **(B)**, China **(C)**, Italy **(D)**, and the United Kingdom **(E)**. Each sector represents the proportion of multi-RP documents affiliated with a given institution, with the remaining institutions collectively categorized as “Others.” Abbreviations: RP, responsible party (corresponding author); multi-RP, documents with more than one corresponding author.

Fifty-four countries contributed to the WoSCC corpus, and the top ten collectively produced 71.5% of total output (Figure 4A). The United States led with 281 publications, closely followed by China (280), Italy (127), and the United Kingdom (76); Canada, Ireland, Korea, Australia, Norway, and France completed the top ten. The Scopus ranking was geographically similar, with the United States (201), China (179), Italy (78), the United Kingdom (56), Ireland (41), Australia (38), Sweden (33), Korea (31), France (30), and Japan (26) as the leading contributors (Supplementary Figure 4). Multiple-country publication (MCP) ratios provided a complementary measure of international collaboration intensity. France (37.2%) and Ireland (30.5%), followed closely by Australia (30.0%), exhibited the highest MCP ratios, whereas China’s MCP ratio was 9.3% (Figure 4A). Article-type distributions within the top-four contributing countries revealed further heterogeneity: both China and the United States produced significantly more research articles than reviews (*p < 0.001*), whereas Italy and the United Kingdom showed no significant difference between article types (Figure 5A).

### Institutional contributions

University College Cork led overall institutional output with 136 WoSCC publications, followed by the University of North Carolina (123) and the University of California System (101), with US institutions occupying six of the top eleven positions (Figure 4B). Country-specific institutional profiles differed markedly in their degree of internal concentration (Figures 5B–E). In the United States, the top-ten institutions accounted for 34.6% of national output, with Mayo Clinic (7.3%), Cedars-Sinai Medical Center (6.0%), and the University of North Carolina (5.1%) leading (Figure 5B). The top-ten Chinese institutions accounted for 24.9% of national output, led by Huazhong University of Science and Technology (3.8%), Zhejiang University (3.5%), and Peking University (2.7%) (Figure 5C). Italy showed an intermediate concentration (top-ten 39.3%), led by the Università Cattolica del Sacro Cuore (11.3%) and the University of Bologna (8.7%) (Figure 5D). The United Kingdom displayed the opposite pattern, with the top-ten institutions accounting for 63.2% of national output and King’s College London alone producing 20.7% of UK publications, followed by St James’s University Hospital (11.5%) and the University of Nottingham Hospital (6.9%) (Figure 5E). Scopus partially corroborated the WoSCC ranking (Supplementary Figure 4). University College Cork again led, although Mayo Clinic and UNC School of Medicine replaced the University of California System and the University of Bergen in the top four. PubMed showed a broader institutional spread (Supplementary Figure 2). The University of Bergen and the University of California System again appeared in the top ten, while the overall ranking was led by the University of Gothenburg and Haukeland University Hospital.

### Institution–keyword–country flow relationships

A Sankey diagram was constructed to visualize the triadic relationships linking institutions, research themes, and national contexts (Supplementary Figure 5). The dominant thematic streams converged on irritable bowel syndrome, gut microbiota, probiotics, and dysbiosis, with secondary flows to fecal microbiota transplantation, visceral hypersensitivity, and the gut–brain axis. Institutional contributions concentrated within a small number of international hubs, including University College Cork, the University of Gothenburg, Cedars-Sinai Medical Center, Mayo Clinic, and several University of California campuses, with visible secondary contributions from European clinical research centers. National-level flows were strongly polarized toward the United States and China on the output side, while displaying substantial cross-regional connectivity among European countries and between Europe and East Asian centers.

### Journal distribution

**Figure 6:**
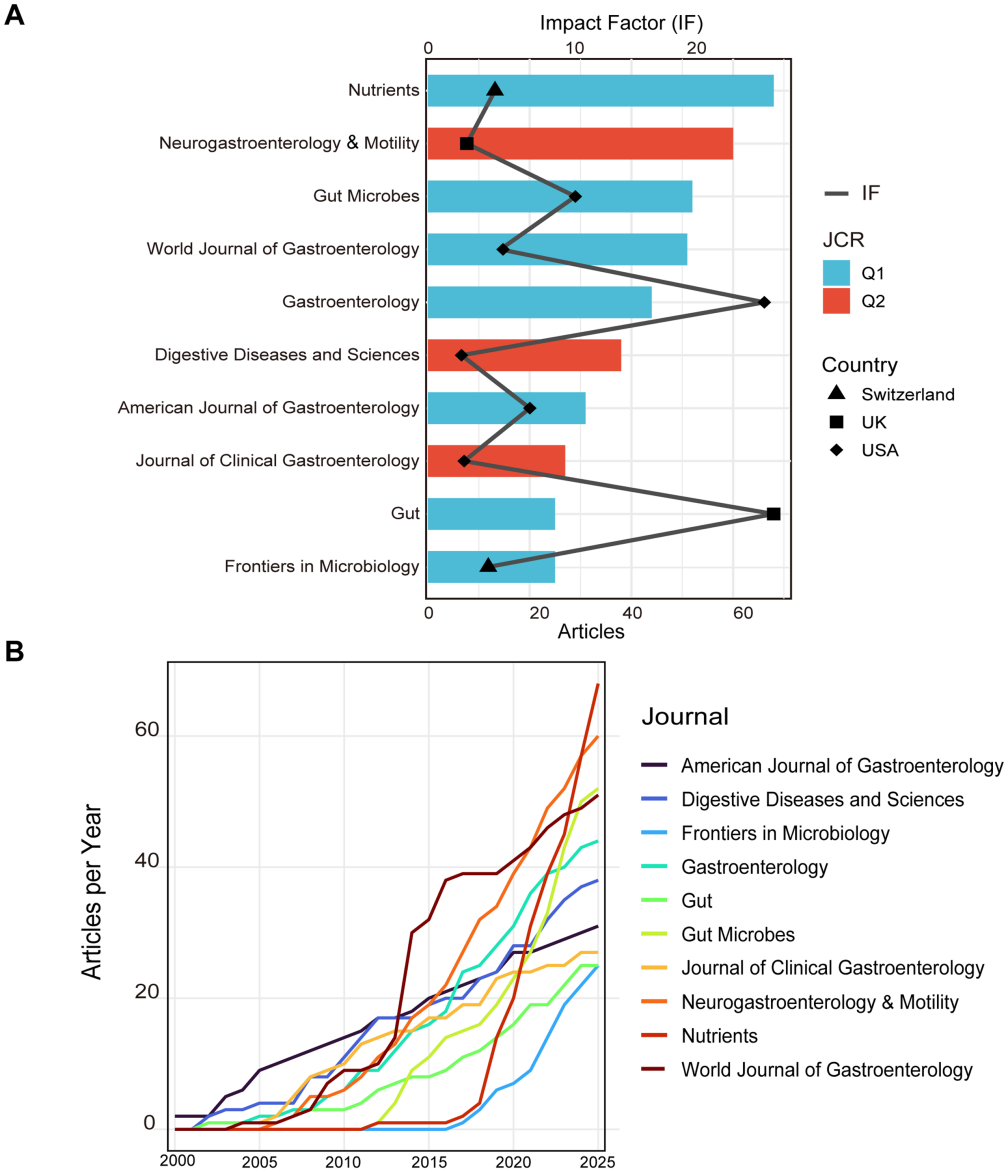
Journal distribution and publication dynamics. **(A)** Top 10 journals publishing studies on IBS and the gut microbiome. Bar charts represent the total number of publications contributed by each journal, with colors indicating Journal Citation Reports (JCR) quartile rankings. The line plot shows the corresponding Impact Factor for each journal. Different shapes denote the geographic location of the journal publishers. **(B)** Temporal publication trends of the top 10 journals, illustrating the evolution of their contributions to the IBS–gut microbiome research field over time.

The WoSCC output spanned 443 journals, of which the top 10 accounted for 28.8% of records, all ranked within JCR Q1 or Q2. *Nutrients* (67 articles) and *Neurogastroenterology and Motility* (59) led by volume (Figure 6A). During 2000–2010, publications concentrated in specialty gastroenterology titles. After 2015, *Nutrients, Frontiers in Microbiology*, and *Gut Microbes* absorbed the majority of output growth, reflecting the field’s expansion into nutrition- and microbiome-focused outlets (Figure 6B). The Scopus journal distribution closely paralleled the WoSCC pattern, with *Nutrients* (56 articles), *Neurogastroenterology and Motility* (48), and *World Journal of Gastroenterology* (33) as the highest-volume outlets. The PubMed ranking was similarly led by *World Journal of Gastroenterology*, *Nutrients*, and *Neurogastroenterology and Motility*, with *Alimentary Pharmacology* and *Therapeutics* and *Gut Microbes* following jointly (Supplementary Figures 6 and 7).

### Author productivity and citation impact

**Figure 7:**
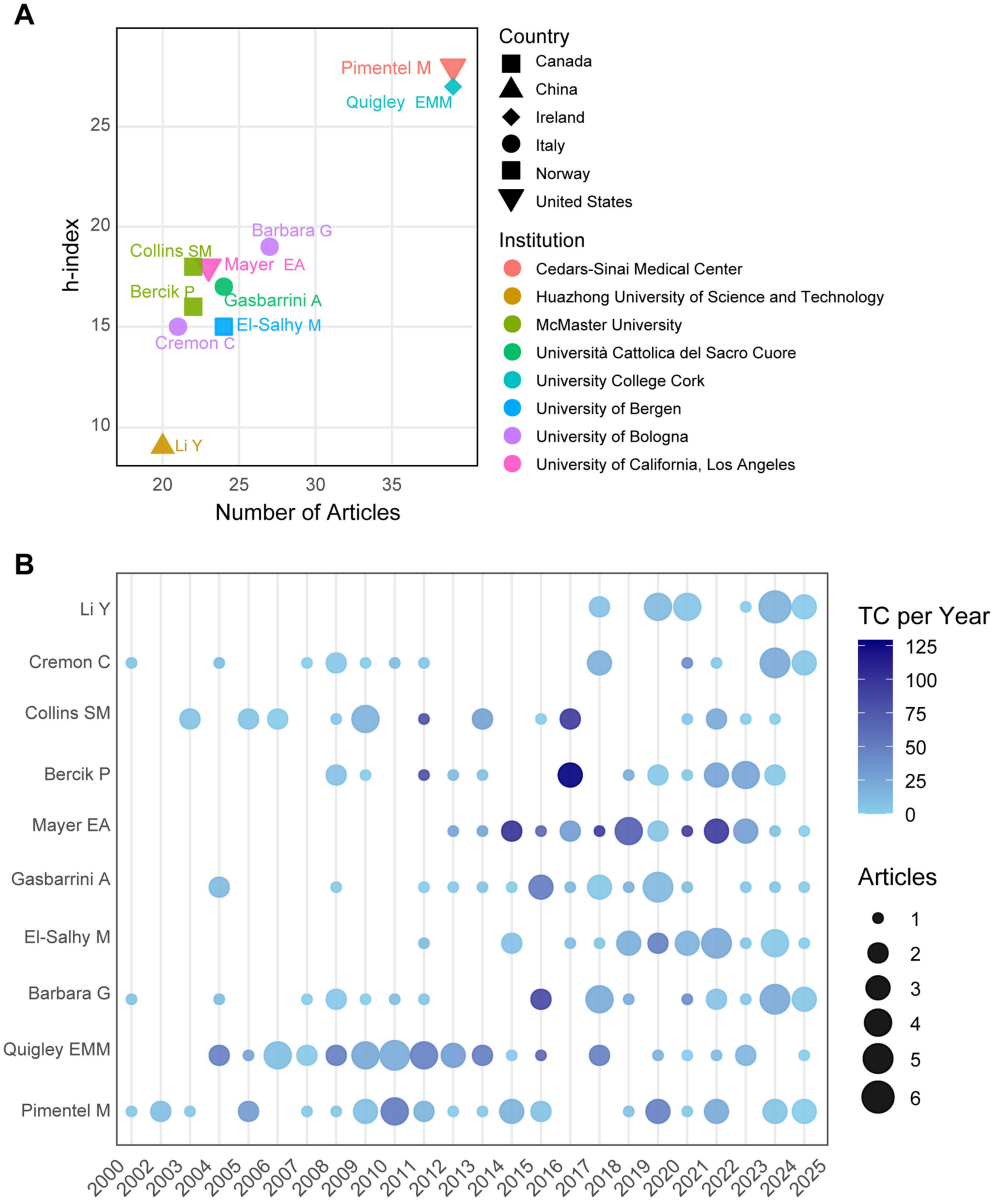
Productivity and impact of leading authors in IBS–gut microbiome research. **(A)** Relationship between publication counts and local h-index among top authors, showing the association between productivity and citation impact within the field. **(B)** Annual publication output and citation intensity of major contributors, illustrating temporal activity patterns and their influence on field development.

Author-level analysis revealed a partial divergence between publication volume and citation impact (Figure 7; Supplementary Table 2). Pimentel M and Quigley EMM jointly led publication output and had the two highest local h-index values. Barbara G ranked next, while Mayer EA and Collins SM had identical local h-index values despite lower publication counts. Gasbarrini A and Bercik P also showed relatively high local h-index values. Temporally, Pimentel M and Quigley EMM maintained continuous output from the early 2000s through 2025. Their activity spanned all three developmental phases identified in this analysis. Bercik P produced the single most prominent citation event in the temporal record during 2016–2017. By contrast, El-Salhy M and Li Y showed output concentrated almost entirely after 2019. These investigators therefore represent prominence emerging within the current phase. All author-level metrics were calculated within the retrieved corpus and reflect within-field prominence rather than career-wide standing. These patterns were largely consistent with Scopus-derived metrics. Quigley EMM and Pimentel M retained their leading positions in publication volume and h-index. The temporal activity profiles of high-output authors also showed no substantive divergence between databases, supporting the robustness of the author-level rankings (Supplementary Figure 8).

### Most cited publications

The ten most cited publications in the WoSCC corpus were published between 2005 and 2019. Local citation counts ranged from 118 to 222, and global citation counts ranged from 336 to 1,064 (Supplementary Table 1). Two broad categories were evident. Foundational profiling and mechanistic studies accumulated the highest global citation counts, reflecting broad interdisciplinary uptake. These included O’Mahony et al. (2005) (191 local, 1,064 global citations, local-to-global ratio [LC/GC] = 17.95%), Rajilić–Stojanović et al. (2011) (222 local, 806 global, LC/GC = 27.54%), and Kassinen et al. (2007) (192 local, 764 global, LC/GC = 25.13%). Intervention- and synthesis-focused studies, including Pittayanon et al. (2019) (LC/GC = 40.25%), Tap et al. (2017) (LC/GC = 39.91%), Tana et al. (2010) (LC/GC = 36.32%), and Carroll et al. (2012) (LC/GC = 35.12%), exhibited substantially higher LC/GC ratios. This pattern is consistent with the tendency of meta-analyses and intervention trials to generate citations mainly within the clinical specialty to which they are most directly relevant.

### Reference citation bursts

Reference burst detection identified publications whose citation frequency rose abruptly during a defined interval. These bursts marked influential anchors across the field’s three developmental phases (Supplementary Figure 9).

Phase I (approximately 2000–2012): Culture-dependent benchmarks and early mechanistic framing. Pimentel (2003) (burst strength 22.74) provided early clinical evidence linking lactulose breath test normalization to symptom improvement, supporting the SIBO-related treatment hypothesis in IBS. O’Mahony et al. (2005) (29.57) provided an immunological bridge between a probiotic intervention and host cytokine correction, integrating microbial exposure with host mucosal biology. Kassinen et al. (2007) (30.66) catalyzed the methodological transition from single-organism enumeration to community-level analysis, making culture-independent profiling the field’s default analytical lens.

Phase II (approximately 2012–2017): Community-level characterization and interventional consolidation. The TARGET 1 and 2 rifaximin trials (Pimentel et al., 2011) (40.32) supplied the first adequately powered demonstration that the microbiome is clinically modifiable at trial scale. Jeffery et al. (2012) (37.91) demonstrated that microbiome-based patient clusters do not correspond to Rome III symptom subtypes. This challenged a central enrollment assumption that had organized a decade of IBS trial design. Halmos et al. (2014) and Halmos et al. (2015) (burst strengths 21.51 and 23.29) established dietary substrate delivery through FODMAP manipulation as a quantifiable modulator of the gut community, linking diet, microbiota, and symptom outcome.

Phase III (approximately 2017–2025): Synthesis and mechanistic consolidation. Pittayanon et al. (2019) (52.65) consolidated the compositional evidence across 24 studies into a reproducible signature of reduced *Lactobacillus* and *Bifidobacterium* alongside increased *Firmicutes*. Tap et al. (2017) (44.03) introduced machine-learning-based classification by linking methanogen abundance and bacterial richness to IBS symptom severity. Sperber et al. (2021) (44.00) anchored the literature to a Rome IV global prevalence framework, and El-Salhy et al. (2020) (25.88) established dose-dependent fecal microbiota transplantation benefit in a placebo-controlled design. The higher raw burst strengths in this phase partly reflect the larger contemporary literature size and should be interpreted alongside active-burst duration.

### Keyword frequency and temporal trends

**Figure 8:**
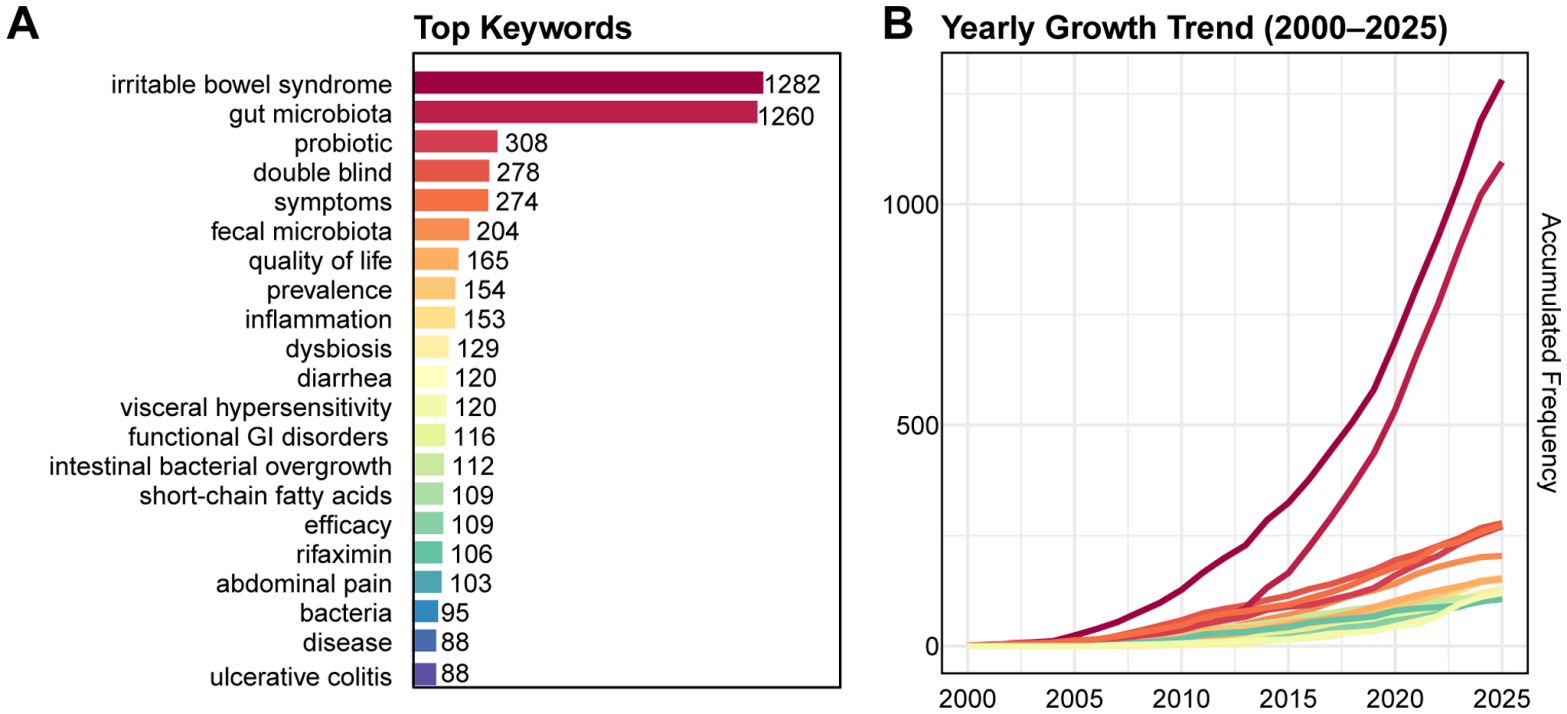
Keyword frequency and temporal trend analysis. **(A)** Distribution of the top 20 high-frequency keywords in the WoSCC dataset (2000–2025). Core terms were dominated by “irritable bowel syndrome” (*n* = 1,282) and “gut microbiota” (*n* = 1,260), reflecting the field’s primary thematic orientation. Intervention- and trial-related terms (“probiotic,” “double blind,” “fecal microbiota,” and “rifaximin”) formed a prominent secondary layer. Symptom- and outcome-related terms (“symptoms,” “quality of life,” “prevalence,” and “abdominal pain”) and mechanistic terms (“inflammation,” “dysbiosis,” “visceral hypersensitivity,” and “intestinal bacterial overgrowth”) were also prominent. **(B)** Accumulated growth trends of the top keywords over time. “Irritable bowel syndrome” and “gut microbiota” showed markedly steeper accumulation than all other terms, with divergence from the remaining keywords becoming pronounced after approximately 2013 and accelerating after 2018.

Descriptive keyword statistics characterized how the field’s vocabulary evolved over time (Figure 8). In WoSCC, the anchor terms irritable bowel syndrome (1,282 occurrences) and gut microbiota (1,260 occurrences) appeared at near-identical frequencies, reflecting the structural co-dominance of the disease descriptor and its molecular correlate. A second tier of high-frequency terms included dysbiosis, probiotics, and fecal microbiota transplantation. Temporal analysis showed that the mechanistic vocabulary related to the gut–brain axis, short-chain fatty acids, and visceral hypersensitivity entered the field at low frequency during the early phase. These terms expanded during the transitional phase and crossed into high-frequency territory only in the most recent phase. Terms characteristic of the earliest period, such as microflora and bacterial overgrowth, receded progressively as the field transitioned from classical microbiology to sequencing-based community profiling. The Scopus keyword frequency distributions reproduced these top-tier patterns (Supplementary Figure 10).

### Keyword co-occurrence network and thematic clusters

Keyword co-occurrence analysis resolved the WoSCC corpus into seven thematic clusters (Supplementary Figure 11). Four clusters contained the principal high-weight and high total link strength (TLS) nodes most relevant to the field’s main structure. These clusters are examined in detail because they represent the mechanistic, interventional, diagnostic, and dietary axes of IBS–gut microbiota research.

The salmon cluster formed the mechanistic core, anchored by microbiota (weight 276, TLS 2017), inflammation (145, TLS 1051), visceral hypersensitivity (112, TLS 782), and gut–brain axis (58, TLS 392), alongside stress, anxiety, depression, serotonin, barrier function, and immune activation. The concentration of inflammatory, neuropsychological and gut–brain terms within this cluster, together with metabolite-related terms in the adjacent microbiome-centered cluster, suggests that microbiota, neuroimmune signaling, and visceral hypersensitivity remain addressed within shared conceptual and methodological contexts. The violet cluster consolidated interventional vocabulary centered on probiotics (263, TLS 2087), double-blind (263, TLS 2074), efficacy (104, TLS 823), and placebo-controlled trial (67, TLS 509), with co-occurring microbial genera including *Lactobacillus* and *Bifidobacterium*. This pattern is consistent with a literature in which probiotic and other microbiota-directed interventions have become increasingly standardized through controlled clinical designs.

The gold cluster grouped diagnostic and clinical IBS vocabulary, anchored by irritable bowel syndrome (696, TLS 4900, the highest-weight node in the network), symptoms (263, TLS 1905), intestinal bacterial overgrowth (110, TLS 857), and rifaximin (104, TLS 790). Together with dysbiosis in the adjacent microbiome-centered cluster, this structure preserves the longstanding parallel framing of IBS–microbiome research around symptom-defined disease, luminal microbial imbalance, and pharmacological modulation. The pink cluster represented the dietary and patient-outcome axis, centered on gut microbiota (435, TLS 2954), quality-of-life (145, TLS 1142), dysbiosis (127, TLS 907), short-chain fatty acids (99, TLS 670), and low FODMAP diet (33, TLS 271). This cluster shows where microbiome composition, microbial metabolites, dietary intervention, and patient-reported outcomes converge.

The remaining clusters further delineated the field’s thematic structure. Infection-related mucosal dysfunction remained visible through post-infectious IBS, bacterial gastroenteritis, permeability, barrier function, and immune activation. The blue cluster centered on molecular and community profiling terms such as intestinal microbiota, fecal microbiota, molecular analysis, metabolomics, and metagenomics, highlighting the continued role of sequencing and multi-omics approaches. FMT and inflammatory bowel disease-related terms formed a broader therapeutic microbiota context, while prevalence, efficacy, severity, and validation remained important contexts for evaluating disease burden, symptom-directed therapy, and validation of microbiota-focused findings.

### Keyword citation bursts

Keyword burst detection provided fine-grained markers of the field’s evolving attention (Supplementary Figure 12). The earliest bursts centered on culture-based luminal microbiology and early interventional designs, with intestinal bacterial overgrowth (14.49, 2003–2016) and randomized controlled trial (16.10, 2006–2017) recording the highest strengths of this phase. They were accompanied by bacterial gastroenteritis, bacterial overgrowth, breath test, and rifaximin, consistent with the period’s dominant agenda of testing the SIBO hypothesis within formal trial frameworks. A subsequent transitional cluster marked the shift toward sequencing-enabled community profiling. Gastrointestinal microbiota (10.93, 2010–2015) and fecal microbiota (6.74, 2010–2013) signaled rapid methodological displacement of culture-based approaches, while the emergence of anxiety-like behavior (7.15, 2011–2017) coincided with the early emergence of gut–brain axis vocabulary. Among currently active bursts, visceral hyperalgesia (13.93, 2022–2025) and hypothalamic–pituitary–adrenal axis dysregulation (13.12, 2023–2025) represent the two highest burst strengths recorded among keywords with end dates extending to the study endpoint, alongside dysbiosis (7.28, 2021–2025) and metabolism (6.54, 2022–2025). Together, these bursts identify microbiota–neuroimmune interactions as an active conceptual frontier.

To facilitate rapid cross-reference and to complement the network and temporal visualizations, Supplementary Table 3 summarizes selected key bibliometric metrics from the primary WoSCC dataset, including corpus size, publication trends, leading countries, collaboration ratios, institutions, journals, authors, and major keyword/burst indicators, with brief cross-database support from Scopus and PubMed where applicable.

## Discussion

Mapping IBS–gut microbiome research from 2000 to 2025 across three independently indexed databases reveals a literature shaped by sequential methodological and conceptual transitions. Annual output grew from fewer than ten publications per year before 2010 to 162 in 2025. This acceleration was reproduced across WoSCC, Scopus, and PubMed, while institutional and journal rankings showed platform-dependent variation. The United States and China accounted for the largest share of global productivity. Within several leading countries, a small number of institutions concentrated a disproportionate fraction of national output. All ten highest-output journals were ranked Q1 or Q2 in JCR. Together, these structural features describe a rapidly expanding field that now spans clinical gastroenterology, microbiology, and nutritional science. At the same time, fundamental questions remain unresolved, particularly the causal role of dysbiosis in IBS and the translational value of compositional findings.

More than half of the field’s cumulative output occurred during the most recent developmental phase. This concentration reflects the convergence of technological, institutional, and conceptual drivers whose timing was not simultaneous. Illumina-based 16S rRNA gene sequencing reached per-sample costs compatible with standard research budgets by the early 2010s (Caporaso et al., 2012). However, the keyword record indicates that sequencing terminology entered the IBS literature with a lag of nearly a decade. This pattern suggests that clinical uptake was limited not only by cost but also by study design. Community-level microbial data had to be incorporated into symptom-based trial frameworks before they could be used at scale. The Human Microbiome Project reference publications of 2012 then provided methodological standards and reference cohorts that IBS studies could adopt directly (The Human Microbiome Project Consortium, 2012), while the 2013 Rome Foundation microbiome report (Simrén et al., 2013) and the 2016 Rome IV reclassification of IBS as a disorder of gut-brain interaction (Lacy et al., 2016) redirected attention toward microbiome-adjacent mechanisms, with the full effect apparent in publication volumes from approximately 2019 onward. The emergence of Tap et al. (2017) as a burst reference in 2017 marked a further methodological inflection. At that point, machine-learning-based severity classification from microbiota profiles began to match the analytical ambition to the scale of generated data.

The geographic architecture of the field was dominated by the United States and China in terms of publication volume. The low multinational collaboration ratio for China (9.3%) should not be interpreted as a straightforward indicator of collaborative reluctance. Large domestic patient cohorts may reduce the practical incentive for international recruitment because adequate sample sizes can often be achieved without cross-border collaboration. A further consideration is that a portion of Chinese clinical research is published in regional journals outside the primary coverage of WoSCC and Scopus, which may cause the observed MCP ratio to underestimate the true extent of Chinese participation in internationally oriented work (Mongeon and Paul-Hus, 2016). Conversely, the elevated multinational collaboration ratios observed for France (37.2%), Ireland (30.5%), and Australia (30.0%) probably reflect structural opportunity as well as collaborative disposition: investigators in countries with smaller domestic patient populations have stronger incentives to seek international partnerships that larger research ecosystems do not encounter to the same degree. Among the country-level findings, the United Kingdom displayed a particularly concentrated output structure. Its top ten institutions accounted for 63.2% of national publications, consistent with the centralization of UK biomedical infrastructure around a small number of established academic medical centers.

These country-level patterns should also be interpreted against the broader funding geography of microbiome science. A bibliometric overview of gut microbiome research in human health and disease reported marked growth in funded microbiome publications, funding agencies, and funded projects, with funded articles accounting for a substantial proportion of the field in recent years (Huang et al., 2022). This broader funding context is consistent with the observed distinction between total publication volume and collaboration intensity. Large national research ecosystems, particularly those in the United States and China, may be better positioned to generate high absolute output. By contrast, smaller but programmatically organized systems, such as Ireland, may achieve higher international collaboration ratios and greater institutional concentration. These patterns suggest that publication volume, international collaboration, and institutional dominance reflect related but analytically distinct dimensions of the IBS–microbiome research landscape.

The institutional ranking yielded one of the study’s more striking observations: University College Cork led global output with 136 WoSCC publications, substantially exceeding institutions embedded in much larger national research systems. This prominence is structurally coherent when viewed against its institutional context. University College Cork houses the APC Microbiome Ireland program, one of the earliest and most sustained dedicated microbiome research centers globally, which has concentrated specialized expertise, longitudinal cohort infrastructure, and a core of senior investigators in the IBS–microbiome field over more than two decades (Cryan et al., 2019). Ireland’s high multinational collaboration ratio further suggests that this output was produced within an internationally integrated research network rather than a domestically insular program. In contrast, Chinese and US output was distributed far more broadly across larger and more heterogeneous national research bases, with the top ten institutions accounting for 24.9 and 34.6% of respective national output. Within the United States, no single institution dominated the ranking. Output was distributed across Mayo Clinic, Cedars-Sinai Medical Center, and several University of California campuses, reflecting integration into a specialized multi-center clinical research network.

Publication channels shifted progressively over the study period, reflecting the field’s expanding disciplinary reach. During the first decade, output was concentrated in specialty gastroenterology titles, including *Neurogastroenterology and Motility* and *Alimentary Pharmacology and Therapeutics*, consistent with a literature primarily oriented toward clinical characterization and pharmacological intervention. From approximately 2015 onward, *Nutrients*, *Frontiers in Microbiology*, and *Gut Microbes* absorbed a substantial share of output growth. IBS–microbiome research therefore became increasingly integrated into broader microbiome and nutritional science. The concentration of output in high-ranking venues across both traditional gastroenterology titles and newer microbiome and nutrition outlets reflects disciplinary broadening into adjacent scientific communities.

Author-level analysis revealed that investigators with the highest publication counts, including Pimentel M and Quigley EMM, built their prominence through sustained programs of sequential clinical evidence generation, exemplified by the rifaximin trial series. High aggregate citation volumes, by contrast, were concentrated among investigators whose contributions were primarily conceptual, including Dinan TG and Cryan JF (Supplementary Table 2), whose gut–brain axis program achieved broad cross-disciplinary uptake beyond the IBS–microbiome field itself. This dual structure reflects the coexistence of clinical and mechanistic research trajectories. Publication counts and citation impact therefore capture different dimensions of scientific contribution and should be interpreted as complementary rather than equivalent.

The structural patterns described above capture the field’s current architecture, but understanding how that architecture was built requires tracing the sequence of conceptual and methodological shifts that produced it. Reference and keyword citation burst analyses identify three successive developmental phases, each defined by a distinct set of dominant questions, available tools, and unresolved problems that shaped the agenda of the next phase.

The earliest developmental phase, spanning roughly 2000 to 2012, was bounded by the technical repertoire of its time. Culture-based enumeration and single-organism hypotheses structured the analytical vocabulary of the period, and the landmark contributions of this period bear the marks of those constraints. Pimentel et al. (2000) positioned small intestinal bacterial overgrowth at the center of IBS pathophysiology. This work established the antibiotic-based intervention paradigm that would organize trial design for the subsequent decade. O’Mahony et al. (2005) provided the first immunological bridge between probiotic intervention and host cytokine correction. Kassinen et al. (2007) catalyzed the methodological transition from single-organism enumeration toward community-level compositional profiling. These were substantive advances, but the cross-sectional case–control architecture that these studies employed became the field’s default study design, and this methodological legacy has constrained causal inference through all subsequent phases. A parallel unresolved question concerns the biological status of small intestinal bacterial overgrowth. It remains unclear whether SIBO is a distinct pathophysiological entity, a downstream consequence of impaired motility, or a diagnostic artifact of breath-testing methodology (Pimentel et al., 2011; Pimentel et al., 2000; Pimentel et al., 2020). This ambiguity continues to complicate the interpretation of antibiotic-based intervention data.

The transitional phase, spanning approximately 2012 to 2017, generated the field’s most substantive conceptual progress. It was also the period in which the gap between discovery and trial implementation became most apparent. Rifaximin trials demonstrated at adequate statistical power that the gut microbiome is a clinically modifiable variable (Pimentel et al., 2011). Halmos et al. (2014) and Halmos et al. (2015) established dietary FODMAP manipulation as a quantifiable modulator of microbial composition. Their work connected dietary substrate delivery, community-level response, and symptom outcome, a finding later supported by systematic review evidence (So et al., 2022). Jeffery et al. (2012) produced what may be the period’s most important and underutilized finding, demonstrating that Rome III symptom subtypes do not correspond to microbial phenotypes and that an alternative species-level stratification identifies biologically distinct patient subsets. The implications of this finding were not incorporated into subsequent trial design. IBS enrollment continued to rely on Rome IV symptom criteria (Vasant et al., 2021), reproducing the stratification mismatch that Jeffery et al. had identified. Candidate alternatives, including microbiota-based clustering, fecal short-chain fatty acid profiling, and intestinal permeability indices, were proposed but not prospectively validated as enrollment criteria (Sun et al., 2019; Camilleri, 2019). This translational failure compounded an existing analytical problem. Placebo response rates in IBS trials are estimated at 27 to 40% (Bosman et al., 2021), compressing the observable treatment window regardless of biological activity. This makes subtle microbial modulatory effects difficult to detect against symptom-based endpoint variance. Whether the modest effect sizes reported in rifaximin and probiotic trials (Ford et al., 2014) reflect genuine biological heterogeneity, stratification along the wrong axis, or the inherent limitations of symptom-based endpoints remains unresolved.

The most recent phase, extending from approximately 2017 to 2025, has shifted from compositional synthesis toward mechanistic interrogation of neuroimmune and neuroendocrine pathways. Pittayanon et al. (2019) consolidated compositional evidence across 24 studies into a reproducible group-level signature. Tap et al. (2017) and Tap et al. (2021) extended this synthesis through machine-learning-based severity classification from microbiota profiles. Mars et al. (2020) provided the first longitudinal multi-omics demonstration of mechanistically distinct patient subsets, and El-Salhy et al. (2020) provided placebo-controlled randomized evidence of dose-dependent fecal microbiota transplantation benefit. Zhao et al. (2022) subsequently synthesized randomized trial evidence, while Talley and Irani (2020) provided a clinical commentary on these outcomes. The strongest currently active keyword bursts converge on visceral hyperalgesia and hypothalamic–pituitary–adrenal axis dysregulation, and independent mechanistic work has begun to characterize the candidate pathways underlying this convergence. These include enterochromaffin-cell serotonin synthesis modulated by short-chain fatty acids (Reigstad et al., 2015; Yano et al., 2015), mast-cell activation downstream of barrier disruption (Barbara et al., 2004), tryptophan catabolism through serotonergic and kynurenic pathways (Kennedy et al., 2017), and microbiota-dependent modulation of stress-axis reactivity (Sudo et al., 2004; Foster et al., 2017). Three outstanding questions will determine whether this mechanistic frontier generates clinical impact commensurate with its bibliometric prominence. First, the causal direction of dysbiosis in IBS remains unestablished. Post-infectious IBS provides the clearest partial evidence for a primary causal role of microbial perturbation (Spiller and Garsed, 2009; Sundin et al., 2015), but meta-analytic evidence has shown that compositional differences between IBS patients and controls replicate poorly across independent cohorts (Pittayanon et al., 2019; Duvallet et al., 2017). Group-level signatures may therefore partly reflect cohort-specific confounding rather than reproducible disease markers. Second, the tension between converging group-level compositional evidence and diverging individual-level mechanistic heterogeneity, documented by Mars et al. (2020), has not been resolved at the trial-design level. The cross-sectional design legacy from the earliest phase remains a structural obstacle to causal inference that cannot be overcome by increases in sample size alone. Third, the gap between the evidentiary maturity of fecal microbiota transplantation and the uncharacterized determinants of donor-response variability remains notable. The super-donor phenomenon (Wilson et al., 2019) and emerging stool-banking standards (Cammarota et al., 2019) suggest that donor characterization represents a tractable near-term target that recipient stratification alone cannot substitute (Mazzawi, 2022).

Dietary modulation has emerged as an important translational direction within IBS–microbiome research. Diet constitutes the primary modifiable determinant of colonic microbial community structure, and the low FODMAP paradigm has provided proof-of-concept that targeted substrate restriction can shift community-level composition with measurable effects on fermentation products and symptom severity. Diet is also embedded within a broader lifestyle–microbiome framework. Recent multi-database bibliometric evidence links prevalent unhealthy lifestyles, including smoking, alcohol consumption, sleep disturbance, sedentary behavior, and high-sugar dietary patterns, to gut microbiota dysbiosis through inflammatory, oxidative-stress, metabolic, and immune-related pathways (Zhou et al., 2026). This broader lifestyle perspective is relevant to IBS because diet–microbiome interactions may influence gut–brain signaling and symptom generation (Sanz et al., 2025). However, dietary protocols are currently assigned based on symptom subtype rather than microbial phenotype, and individual variation in FODMAP-responsive microbial signatures is sufficiently large that population-averaged microbiome effects mask clinically meaningful responder–non-responder distinctions (Vervier et al., 2022; Zhang and Su, 2025). Converting the observational diet–microbiome–symptom evidence base into actionable nutritional recommendations will require prospective trials that incorporate participant-level microbiota profiling as both a stratification variable and a mechanistic outcome measure.

### Limitations

Several methodological limitations should be acknowledged. Restriction to English-language publications may underestimate contributions from non-Anglophone research environments, particularly during the early years when international indexing coverage was more limited. Although legacy descriptors such as flora and microflora were included, some early studies may still have been missed. Citation lag may also lead to underrepresentation of the 2024–2025 literature in burst analyses. In addition, the steep rise in output during the 2017–2025 phase should not be interpreted solely as an IBS–microbiome-specific signal. Although this period coincided with field-specific developments in sequencing-based microbiome profiling and gut–brain research, it also overlapped with wider systemic expansion in scientific publishing. Recent quantitative evidence has highlighted increasing strain and growth across the scholarly publishing ecosystem, particularly in biomedical and multidisciplinary venues (Hanson et al., 2024). Therefore, the proposed phase model should be interpreted in the context of both discipline-specific developments and wider changes in scholarly communication, and current keyword bursts should be viewed as indicators of emerging rather than definitive research directions.

## Conclusion

The IBS–gut microbiome literature has moved through three developmental phases, from culture-dependent foundations through a symptom-microbiome stratification mismatch, to a current mechanistic frontier integrating neuroendocrine pathways and multi-omics. Translating this trajectory into clinical impact will require prospective longitudinal designs for causal inference, randomized trials testing microbiota-based enrollment criteria, fecal microbiota transplantation programs with formalized donor characterization, and dietary intervention trials that embed participant-level microbiota profiling as both a stratification variable and a mechanistic readout.

## Data Availability

The data supporting the findings of this study are included in the article and Supplementary materials. The R scripts used for bibliometrix-based processing, statistical testing, and visualization, together with CiteSpace settings and VOSviewer parameter configurations, are available from the corresponding author upon reasonable request.
