# Supplementary material for: Mapping research trends in irritable bowel syndrome and the gut microbiome: a cross-database bibliometric analysis
Source: Front Microbiol. 2026 Jul 22;17:1900159. doi: 10.3389/fmicb.2026.1900159 (PMC13438131; doi:10.3389/fmicb.2026.1900159)
Supplement: Supplementary file 1 [file Data_Sheet_1.zip › Supplementary_Material_revised.docx]

Supplementary Material

**Supplementary Figures**

**Supplementary Figure 1.** Global publication trends and corresponding-author distribution based on Scopus data. (A) Annual and cumulative publication output from Scopus during the study period. (B) Distribution of corresponding-author countries based on Scopus records.

**Supplementary Figure 2.** Global publication trends and institutional distribution based on PubMed data. (A) Annual and cumulative publication output from PubMed during the study period. (B) Total publication output and temporal accumulation trends of the top 10 affiliations based on PubMed records.

**Supplementary Figure 3.** Article types and authorship characteristics based on Scopus data. (A) Temporal trends in the annual output of original articles and reviews. (B) Comparison of author numbers between document types. (C, D) Distribution of author numbers stratified by journal impact factor group for original articles (C) and reviews (D). ns, not significant; *****p* < 0.0001.

**Supplementary Figure 4.** Research output and impact of top countries and institutions based on Scopus data. (A) Publication volume, international collaboration, and citation impact of the top productive countries. (B) Total publication output and temporal accumulation trends of the top productive institutions.

**Supplementary Figure 5.** Three-field plot showing relationships among institutions (AU_UN), keywords (DE), and countries (AU_CO) based on Web of Science Core Collection data.

**Supplementary Figure 6.** Journal distribution and publication dynamics based on Scopus data. (A) Top productive journals based on Scopus data. Bars represent publication volume, and the line indicates journal impact factor. Bar colors indicate JCR quartiles, and shapes indicate the publisher's country. (B) Annual publication growth trends of the top productive journals.

**Supplementary Figure 7.** Journal distribution and publication dynamics based on PubMed data. (A) Top productive journals based on PubMed data. Bars represent publication volume, and the line indicates journal impact factor. Bar colors indicate JCR quartiles, and shapes indicate the publisher's country. (B) Annual publication growth trends of the top productive journals.

**Supplementary Figure 8.** Scientific production and impact of top authors based on Scopus data. (A) Relationship between the number of publications and local H-index among leading contributors. (B) Annual publication output and citation intensity (total citations per year) of leading authors over time.

**Supplementary Figure 9.** Top 25 references with the strongest citation bursts based on Web of Science Core Collection data. Red bars indicate the periods during which each reference experienced a citation burst, and burst strength reflects the intensity of increased citation attention.

**Supplementary Figure 10.** Keyword frequency and temporal trend analysis based on Scopus data. (A) Distribution of high-frequency keywords identified from Scopus records. (B) Accumulated growth trends of the leading keywords over time.

**Supplementary Figure 11.** Keyword co-occurrence analysis and visualization based on Web of Science Core Collection data. The keyword co-occurrence network illustrates relationships among frequently used keywords. Node size represents keyword frequency, and links indicate co-occurrence relationships among keywords. Only keywords meeting the visualization threshold are displayed.

**Supplementary Figure 12.** Top 25 keywords with the strongest citation bursts based on Web of Science Core Collection data. Red bars indicate the periods during which each keyword experienced a citation burst, and burst strength reflects the intensity of increased research attention.

**Supplementary Table**

**Supplementary Table 3.** Concise summary of selected key bibliometric metrics from the primary Web of Science Core Collection (WoSCC) dataset, with brief cross-database support from Scopus and PubMed where relevant.

| **Domain** | **WoSCC findings** | **Cross-database validation** |
| --- | --- | --- |
| Corpus | 1,502 records (2000–2025) | Scopus: 1,163; PubMed: 975 |
| Publication trend | Peak output: 162 publications (2025); 53.3% of publications were produced during 2017–2025 | Similar acceleration observed in Scopus and PubMed |
| Countries | USA (281), China (280), Italy (127), UK (76) | Same leading-country pattern |
| International collaboration | France (37.2%), Ireland (30.5%), and Australia (30.0%) had the highest multiple-country publication (MCP) ratios | Consistent distinction between productivity and collaboration |
| Institutions | University College Cork (136), University of North Carolina (123), University of California System (101) | Similar institutional leadership |
| Journals | Top 10 journals: 28.8%; *Nutrients* (67); *Neurogastroenterology & Motility* (59) | Similar core journals identified |
| Authors | Quigley EMM; Pimentel M; Barbara G; Mayer EA; El-Salhy M | Comparable author rankings |
